# Serum SARM1 Levels and Diabetic Peripheral Neuropathy in Type 2 Diabetes: Correlation with Clinical Neuropathy Scales and Nerve Conduction Studies and Impact of COVID-19 vaccination

**DOI:** 10.3390/vaccines12020209

**Published:** 2024-02-17

**Authors:** Moafaq S. Alrawaili, Ahmad R. Abuzinadah, Aysha A. AlShareef, Emad A. Hindi, Ahmed K. Bamaga, Weam Alshora, Hashim Sindi

**Affiliations:** 1 Department of Neurology, Faculty of Medicine, King Abdulaziz University, Jeddah 21589, Saudi Arabia; 2 Neuromuscular Medicine Unit, King Abdulaziz University Hospital, King Abdulaziz University, Jeddah 21589, Saudi Arabia; 3 Department of Clinical Anatomy, Faculty of Medicine, King Abdulaziz University, Jeddah 21589, Saudi Arabia; 4 Neurology Unit, Pediatric Department, Faculty of Medicine, King Abdulaziz University, Jeddah 21589, Saudi Arabia; 5 Department of Family Medicine, King Abdulaziz University Hospital, Jeddah 21589, Saudi Arabia; 6 Department of Laboratory Medicine, King Abdulaziz University Hospital, Jeddah 21589, Saudi Arabia

**Keywords:** type 2 diabetes mellitus, diabetic neuropathy, sterile-a and Toll/interleukin-1 receptor domain-containing protein 1, T2DM patients, nerve conduction, clinical neuropathy scales, COVID-19 vaccine

## Abstract

Patients with peripheral neuropathy with type 2 diabetes mellitus (T2DM) are more likely to have functional impairments. Recently, the gene for serum sterile alpha and toll/interleukin receptor motif-containing protein 1 (SARM1), which may contribute to the pathogenesis of Wallerian degeneration, was discovered in mice models of peripheral neuropathy. We set out to assess serum SARM1’s activity as a potential biomarker for the early identification of diabetic peripheral neuropathy in T2DM patients while also examining the impact of the COVID-19 vaccine on SARM1 levels. We assessed the cross-sectional relationships between the SARM1 biomarker, clinical neuropathy scales, and nerve conduction parameters in 80 participants aged between 30 years and 60 years. The analysis was carried out after the patients were split into two groups since we discovered a significant increase in SARM1 levels following the second dose of the COVID-19 vaccination, where group A received one dose of the COVID-19 vaccine inoculation, and group B received two doses of the COVID-19 vaccine. SARM1 was correlated significantly (*p* < 0.05) with MNSIe and NSS in group A and showed a consistent positive correlation with the other neuropathy clinical scales in group A and group B without reaching statistical significance. Additionally, SARM1 was negatively correlated significantly (*p* < 0.05) with the median sensory amplitude in group A and showed a consistent negative correlation with the six other sensory and motor nerves’ potential amplitude in group A and group B without reaching statistical significance. In conclusion, SARM1 showed a consistent correlation with clinical neuropathy scales and nerve conduction parameters after accounting for the influence of COVID-19 vaccination doses.

## 1. Introduction

Due to inadequate insulin production and activity, diabetes mellitus is a metabolic condition that is characterized by abnormally high blood sugar levels [1]. Diabetes is associated with an increased risk of complications and mortality compared with the general population [2]. More than 50% of older adults with type 2 diabetes mellitus (T2DM) are diagnosed with diabetic peripheral neuropathy (DPN) [3]. In Saudi Arabia, DPN is prevalent in 37.4% of patients diagnosed with T2DM [4]. Due to increased amputation and foot ulceration, changes in stride and posture, and higher fall risks, DPN is one of the major causes of disability in people with T2DM [3]. Numerous acute and chronic neuropathy symptoms are brought on by diabetes. The Toronto Panel defines DPN as a symmetrical length-dependent sensory-motor polyneuropathy caused by metabolic alterations in the microvessels owing to chronic hyperglycemia and related cardiovascular risk factors [3]. Despite the poorly understood mechanisms underlying DPN, DPN may be caused by high blood glucose levels that promote the demyelination of nerve fibers, eventually leading to poor neurotransmission and nerve damage [5]. This occurs through the hyperactivity of the polyol pathway owing to decreased insulin secretion and insulin resistance. The rate-limiting enzyme, aldose reductase, converts glucose into sorbitol via the oxidation of nicotinamide adenine dinucleotide phosphate (NADP) to NADP^+^. Sorbitol undergoes further oxidation to fructose by sorbitol dehydrogenase and the reduction of NAD^+^ to NADH^+^. This causes the accumulation of cofactors, such as NADP and NAD, thus leading to a decrease in glutathione levels, which causes oxidative stress and nerve damage [6]. Aldose reductase inhibitors can prevent the onset of DPN in animal models [7]. Oxidative and nitrosative stress pathways are also implicated in the pathophysiology of DPN [8]. Oxidative stress causes the auto-oxidation of glucose, leading to the increased intracellular formation of advanced glycation end products, the activation of diacylglycerol and protein kinase C isoforms, the alteration of mitochondrial function, and increased activity of the hexosamine pathway, which can cause the demyelination and apoptosis of neuronal cells [9,10]. The poly (ADP-ribose) polymerase (PARP) pathway is also implicated in the pathophysiology of DPN. Researchers have observed PARP activation in diabetes, which can cause endothelial cell dysfunction. PARP activation by oxidative stress in the Schwann cells causes peripheral nerve perfusion and conduction deficits, which may be implicated in the pathogenesis of DPN [11]. The symptoms associated with DPN most often originate in the toes and migrate toward the upper limbs. These symptoms include the loss of pain sensation in the affected areas, tingling, burning sensation, allodynia, or hyperalgesia [12]. 

The symptoms of peripheral neuropathy do not indicate the extent of nerve fiber damage, and individuals with severe pain may have minimal sensory deficits. This can be confusing because pain is typically considered a sensory issue, but in peripheral neuropathy, pain does not always correlate directly with the level of nerve damage [13,14].

Axon degeneration is a prominent early feature of several peripheral neuropathies, including DPN [15]. One important advancement in Wallerian degeneration has been the discovery of sterile alpha and toll/interleukin receptor motif-containing protein 1 (SARM1) [16]. Interestingly, Sarm1-/-mice are resistant to streptozotocin-induced diabetes. In addition, Sarm1 gene deficiency significantly reduces DPN in mice; therefore, decelerating axonal degeneration provides a promise for better treatment of this disease [15]. Several studies have reported on an association between SARM1 and DPN in animals; nonetheless, they did not evaluate the serum SARM1 levels in patients with diabetes to confirm its role in the disease. In December 2019, the novel coronavirus, SARS-CoV-2, caused pulmonary-systemic coronavirus diseases (COVID-19), which were first reported in Wuhan, China [17]. Several countries have approved the use of COVID-19 vaccines for human use, providing an important boost to the fight against the disease [18]. These vaccines are comprised of COVID-19 mRNA-based vaccines, viral vector-based vaccines, protein-based vaccines, and attenuated/inactivated virus vaccines [19]. In many cases, severe neurological complications have been reported following the COVID-19 vaccination, including encephalopathy, transverse myelitis, and Bell’s palsy (idiopathic peripheral facial nerve palsy). In addition, Guillain–Barré syndrome has also been reported, and auto-antibody-mediated molecular mimicry is believed to be the cause of post-vaccination nerve damage [20]. The COVID-19 vaccine can cause peripheral and central nervous system complications ranging in severity from minimal to life-threatening [21]. Current research indicates that similar mechanisms may contribute to potential vaccine-induced neuropathies, but this has not been confirmed [22].

This novel study aimed to visualize the serum SARM1 levels in humans and to confirm the hypothesis that serum SARM1 may be closely related to the severity of neuropathy in patients with T2DM as well as the possible effects of COVID-19 vaccination.

## 2. Methodology

### 2.1. Ethical Consideration

The Biomedical Research Committee of the Medical College authorized this investigation (Study Number 176-21\2.exe). Patients have completed the informed consent forms and agreed to participate in the clinical neuropathy assessments, nerve function testing, and biochemical measures.

### 2.2. The Selection of Participants

#### 2.2.1. Inclusion Criteria

The following were the inclusion criteria: (1) Arabic as the native dialect; (2) no other neurological disorders affecting the motor and sensory systems, such as stroke or multiple sclerosis; (3) between the ages of 30 and 60; (4) diabetes diagnosed by a physician and confirmed by a HbA1c level exceeding 6.5%.

#### 2.2.2. Exclusion Criteria

The following were the exclusion criteria:

1. For patients with type 1 diabetes mellitus (T1DM) (on insulin therapy), the reason for excluding patients on insulin therapy (type 1 diabetes mellitus) is to focus specifically on patients with type 2 diabetes for this particular study. T1DM is a distinct form of diabetes that requires lifelong insulin therapy, and the management approaches for type 1 and type 2 diabetes differ significantly. To clarify the exclusion criteria, our study aimed to investigate the impact of the serum levels of SARM1 on neuropathy in individuals with type 2 diabetes.
2. Patients with a history of renal diseases refer to any conditions that affect the kidneys, such as chronic kidney disease, kidney stones, or glomerulonephritis.
3. Patients with a history of hepatic diseases, on the other hand, pertain to disorders that impact the liver, including hepatitis, cirrhosis, or liver cancer.
4. Patients with a history of malignancies encompass various types of cancer, including but not limited to breast, lung, colon, or prostate cancer. Lastly, blood disorders encompass conditions that affect the blood and its components, such as anemia, hemophilia, or leukemia.

These diseases were excluded from our study due to several reasons. Firstly, individuals with renal or hepatic diseases may have an altered metabolism or impaired drug clearance, which could potentially affect the results and interpretation of our study. Additionally, individuals with malignancies or blood disorders often have unique physiological characteristics or undergo treatments that may confound our findings. Finally, our biomarker, SARM1, is expressed in various tissues and organs, including the liver, brain, and kidney. Therefore, any diseases affecting these organs may have an impact on their expression. By excluding these specific conditions, we aimed to ensure a more homogeneous study population and reduce potential confounding factors.

All participants who had not been vaccinated against COVID-19 diseases were excluded according to the Ministry of Health requirements during the global pandemic outbreak of coronavirus. We selected all subjects without knowing their immunization status with the COVID-19 vaccine at the time of selection.

#### 2.2.3. Participant Recruitment

This cross-sectional study was conducted at the King Abdulaziz University Hospital (KAUH), Jeddah, Saudi Arabia, between May 2021 and August 2021. We enrolled a total of 80 participants between the ages of 40 and 60, consisting of 50 patients with T2DM and 30 healthy individuals. These participants were recruited from outpatient clinics, the neuromuscular clinics, and the diabetes clinics at King Abdulaziz University Hospital, Jeddah, KSA. All participants were classified into three major subgroups as follows: diabetic patients with neuropathy (T2DMN) (*n* = 30), diabetic patients without neuropathy (T2DM) (*n* = 20), and healthy participants (HP) (*n* = 30). We encountered difficulties in recruiting a larger number of participants for our research due to the constraints of the COVID-19 pandemic. As a result, our sample size was small. It is important to acknowledge the significant challenges presented by the pandemic in conducting research studies. Taking this context into consideration is crucial for interpreting our results. Additionally, it is worth noting that SARM1 plays a vital role in mediating apoptosis and antiviral immune responses [23]. Given that the majority of COVID-19 vaccines aim to induce an immune response, it is important to conduct a sensitivity analysis to assess the impact of COVID-19 vaccination doses on our study outcomes. The number of vaccine doses is a critical factor in determining the effectiveness and overall impact of our findings. However, this number is subject to uncertainties and potential biases, such as reporting errors, data collection issues, or variations in vaccine distribution strategies. In order to address these concerns, we conducted sensitivity analyses in the two primary research groups, focusing specifically on the number of vaccine doses delivered. The objective of this analysis was to assess the sensitivity of our findings to different scenarios or assumptions regarding the dose count and its effect on serum levels of SARM1. By doing so, we were able to evaluate the robustness of our study’s conclusions and gain insights into the potential impact of variations or uncertainties in the dose count on serum levels of SARM1. Additionally, the sensitivity analysis allowed us to identify influential factors or variables that had a significant effect on the outcomes. This information is valuable for decision-makers and health providers, as it helps them understand the key drivers of the results and make well-informed decisions regarding vaccination strategies.

As a result, we conducted sensitivity analyses in the two primary research groups based on the number of COVID-19 vaccination doses delivered. Group A included participants who had received a single dose, and group B included participants who had received two doses of COVID-19 vaccination. Each study group was subdivided into the following three subgroups:

- Group A included subgroup I, HP (*n* = 18); subgroup II, T2DM (*n* = 10); and subgroup III, T2DMN (*n* = 22).
- Group B included subgroup I, HP (*n* = 12); subgroup II, T2DM (*n* = 10); and subgroup III, T2DMN (*n* = 8).

In order to improve the clarity and transparency of the study’s findings, we requested further information regarding the duration of the vaccine (DV) as well as the duration since the last COVID-19 vaccine administration; it would be beneficial for us to provide a clear explanation of the measure of the DV and its relevance in this research. This would enable readers to have a better understanding of the implications of the results and explore potential correlations with other variables among the different groups in the study.

#### 2.2.4. T2DM Assessment

In addition, all patients with T2DM were formally diagnosed with T2DM based on the American Diabetes Association guidelines.

#### 2.2.5. Diabetic Neuropathy Assessment

Conventionally, diabetic neuropathy was diagnosed and staged by neurologic examinations and nerve conduction studies (NCSs). In this study, we used an MNSI score ≥7.5 to define the diabetic neuropathy group. An MNSI > 7.5 was found to be 90% specific for confirmed DPN [24]. We performed NCS, including the sural nerve sensory potentials amplitude and common peroneal nerve motor potentials amplitude, to grade the severity of neuropathy in patients with or without neuropathy [25,26].

### 2.3. Data Collection and Data Collection Tools

#### 2.3.1. Sample Preparation and the Assessment of Biomarkers

Following an overnight fast (at least 12 h), the participants were admitted as outpatients to the neuromuscular unit at KAUH between 7:30 a.m. and 8:00 a.m. to minimize the effect of diurnal variations. They were instructed to sit quietly for 25 min to 45 min before venipuncture for routine hematology, biochemistry tests, and special testing. During the sample collection, routine biochemical tests were performed in the hematology and biochemistry laboratories. Five milliliters of serum were obtained in a lavender ethylenediaminetetraacetic acid tube, centrifuged at 1500× *g* for 10 min, and stored at −80 °C for subsequent processing. The biochemical parameters included the following parameters: HbA1c, which was measured using the VARIANT II hemoglobin testing system and FBG, which was measured using the SIEMENS biochemistry analyzer. SARM1 was measured by an enzyme-linked immunosorbent assay (ELISA) on a human ELISA reader (Biotek ELX 800). Per the manufacturer’s instructions, the assay was conducted in duplicate (16 plates were measured in duplicate using three control sera to estimate the coefficients of variation) (Bioassay Technology Laboratory ELISA kit, catalog no: E0666Hu, BT LAB Co., Ltd., Shanghai, China).

#### 2.3.2. Diabetic Neuropathy Assessment

We used the Michigan Neuropathy Screening Instrument Arabic version (MNSI-Ar) to measure peripheral neuropathy [24]. This instrument is composed of two independent assessments as follows: (A) the MNSI questionnaire (MNSIq)—a 15-item self-administered questionnaire, where “Yes” responses to questions 1 to 3, 5 to 6, 8 to 9, 11 to 12, and 14 to 15 are counted as 1 point. Questions 4 and 10 are not counted, and questions 7 and 13 count as 1 point if the answer is no. A score of 7 was considered abnormal. The second assessment was a (B) MNSI examination (MNSIe), where a trained healthcare professional assessed each patient. Moreover, we used the Neuropathy Symptoms Score (NSS), Diabetic Neuropathy Symptoms Score (DNS), Modified Toronto Clinical Neuropathy Score questionnaire (mTCNSq), Modified Toronto Clinical Neuropathy Score (mTCNSe), Utah Early Neuropathy Scale examination (UENS), and the Neuropathy Disability Score (NDS). By using multiple scales that assess different aspects of neuropathy, we aimed to identify any discrepancies or variations in the correlation results. The inclusion of multiple neuropathy scales in our study provided a more comprehensive assessment of diabetic neuropathy and ensured the reliability and validity of our findings.

#### 2.3.3. Nerve Conduction Study

We performed the NCS according to the standard protocol [27] using a high-quality NCS machine provided by Natus Medical Incorporated. It was performed bilaterally on all limbs while the participants were lying comfortably in a supine position on the bed. We measured the sensory and motor parameters (latency, amplitude (AMP), duration, and conduction velocity (CV). The neurophysiological profile consisted of the following aspects: (1) motor conduction of the median, ulnar, tibial, and common peroneal nerves, with measurements of the baseline-to-peak AMP (in mV) of the compound muscle action potential, distal motor latency in milliseconds (ms), and motor conduction velocity (in M/s); (2) sensory conduction of the median, ulnar, radial, superficial peroneal, and sural nerves (antidromic and orthodromic techniques), with measurements of baseline-to-peak AMP of the sensory action potential and sensory CV. We required warming up the patients to maintain their skin temperature to the recommended level of 32 °C to 34 °C. The CV was calculated as follows:

CV (M/s) = distance (mm)/latency proximal − latency distal (ms)

### 2.4. Statistical Analyses

All variables were analyzed using descriptive statistics. For the continuous and categorical variables, we computed the median and interquartile ranges (IQR) and the frequencies, respectively. We analyzed the differences between two groups and three or more groups using the nonparametric Mann–Whitney U test (MW-U) and Kruskal–Wallis test (KWT), respectively. Moreover, we performed Spearman’s correlation analysis to examine the relationship between SARM1 expression and various variables. Multinomial logistic regression (MLR) was performed where the T2DMN groups were the dependent variable and the serum SARM1 parameter was the independent variable. Also, we used MLR where the duration of T2DM (years) > 3 groups was the dependent variable and where the serum SARM1 parameter was the independent variable. The confounder factors were adjusted by the number of COVID-19 vaccine doses. Healthy participants were used as the reference group to compute the odds ratio (OR) and 95% confidence interval (CI). Every statistical test was two-sided. The significance threshold was chosen at *p* < 0.05. SPSS v28.0 (Chicago, Armonk, NY, USA) was used for the overall analyses.

## 3. Results

### 3.1. General Characteristics

**Figure 1:**
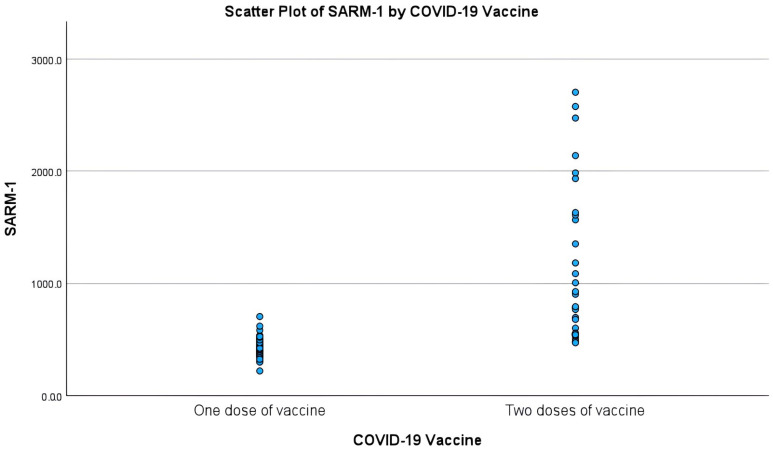
Scatter plot displaying changes in SARM1 levels (ng/L) with the number of COVID-19 vaccine doses.

Of the eighty participants (30 HPs, 20 patients with T2DM, and 30 patients with T2DMN), 18 of these were HPs, 10 patients had T2DM, and 22 patients with T2DMN and had received a single dose of COVID-19 vaccine (group A) and 12 HPs, 10 patients with T2DM and 8 patients with T2DMN have received two doses of COVID-19 vaccine (group B). The gender distribution was similar in the three groups, while the T2DM and T2DMN participants were older than the HP group (Table 1). The disease duration was longer among those with T2DMN compared to T2DM patients. However, the HBA1c% was similar between T2DM and T2DMN participants. The SARM1 level was no different between the three groups. However, after stratifying the analysis based on the number of COVID-19 vaccinations, the SARM1 level was different in group A between the HP, T2DM, and T2DMN participants, while there was no difference in group B; see Figure 1 (Table 1, Table 2 and Table 3). There was a significant influence of the COVID-19 vaccination doses on the SARM1 level, as two doses of vaccination caused a huge surge in the SARM1 compared to a single dose (Figure 1).

### 3.2. Comparison of the Clinical Neuropathy Scores and NCSs

The clinical neuropathy scores (MNSI-Ar, mTCNS, NSS, NDS, UENS) were higher among the T2DMN group compared to the T2DM and the HP groups in the whole cohort and in group A and group B (*p* = 0.000) (Table 1 and Table 2).

The Kruskal–Wallis test for the NCS parameters (including Amplitude (AMP) of median sensory (MS), ulnar sensory (US), sural sensory (SS), and superficial peroneal sensory (SPS) showed a significant decrease in the T2DMN group, compared with the HP group in the whole cohort and in groups A and B (*p* ≤ 0.003), except US AMP in group B, where there was no significant difference (Table 1 and Table 2).

### 3.3. Correlation between SARM1 and Clinical Parameters

Table 4 summarizes the correlation between serum SARM1 and the clinical parameters of all participants. We identified a negative correlation between serum SARM1 with US and RS amplitudes (*p* ≤ 0.05). Table 5 summarizes the correlation between serum SARM1 and the clinical parameters among the groups stratified by the number of previous COVID-19 vaccinations: group A (a single dose of COVID-19 vaccine) and group B (two doses of COVID-19 vaccine). We identified a positive correlation between serum SARM1 with the duration of diabetes (DD), MNSIe, and NSS in group A (*p* ≤ 0.05).

**Figure 2:**
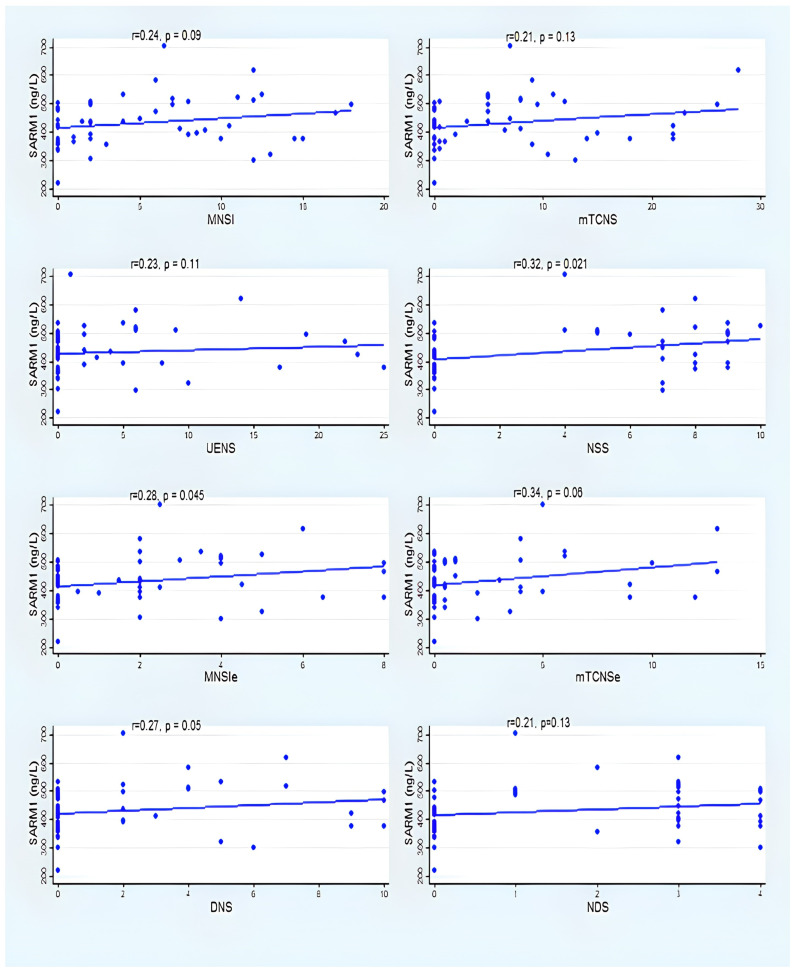
Correlation between SARM1 and neuropathy scales in group A.

**Figure 3:**
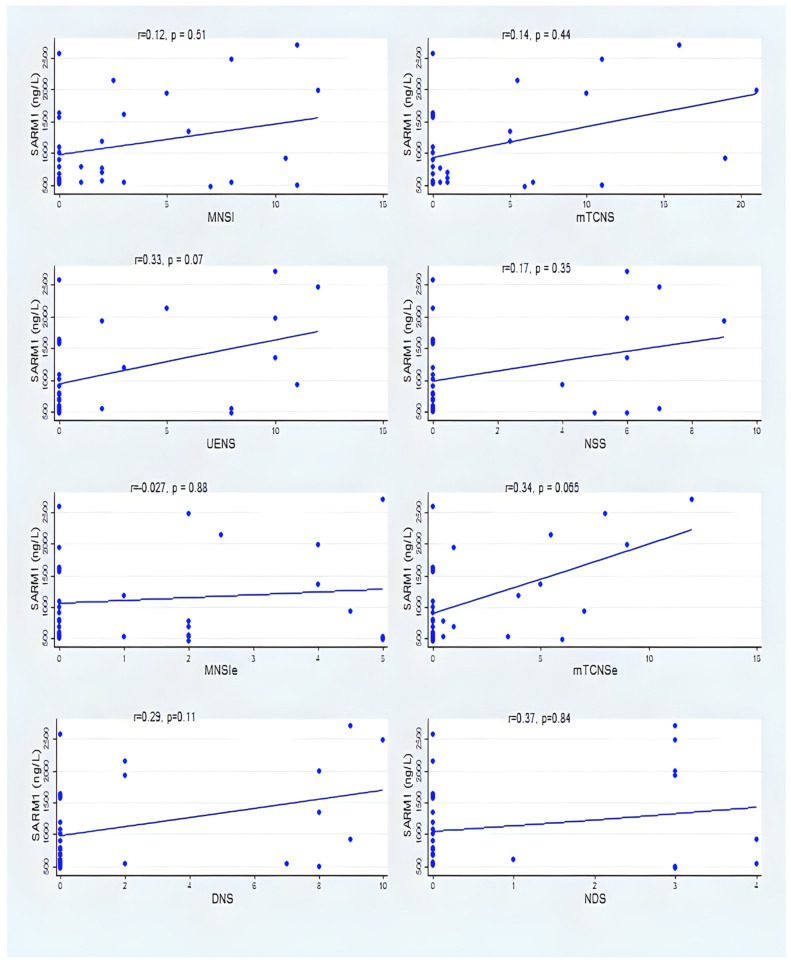
Correlation between SARM1 and neuropathy scales in group B.

Figure 2 and Figure 3 summarize the correlation between serum SARM1 and the clinical neuropathy scales among group A and group B. Despite the absence of a statistically significant correlation between SARM1 and several clinical neuropathy scales (MNSI, mTCNS, UENS, DNS, and NDS), the correlations were consistently in the positive direction in group A and group B (Figure 2 and Figure 3) (see supplementary Tables S5–S8 for details).

**Figure 4:**
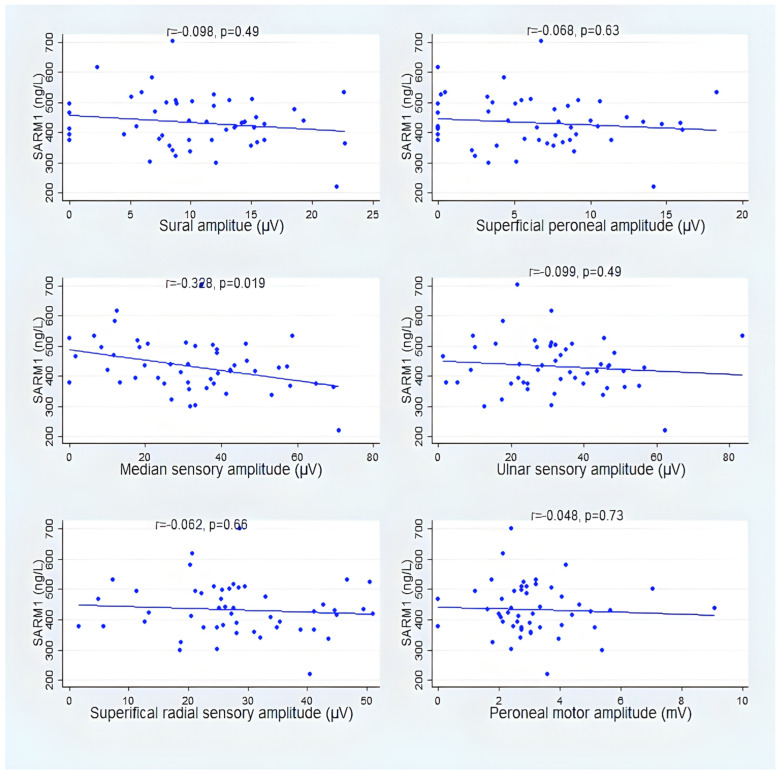
Correlation between SARM1 and amplitude of sensory and motor nerves action potentials in group A.

**Figure 5:**
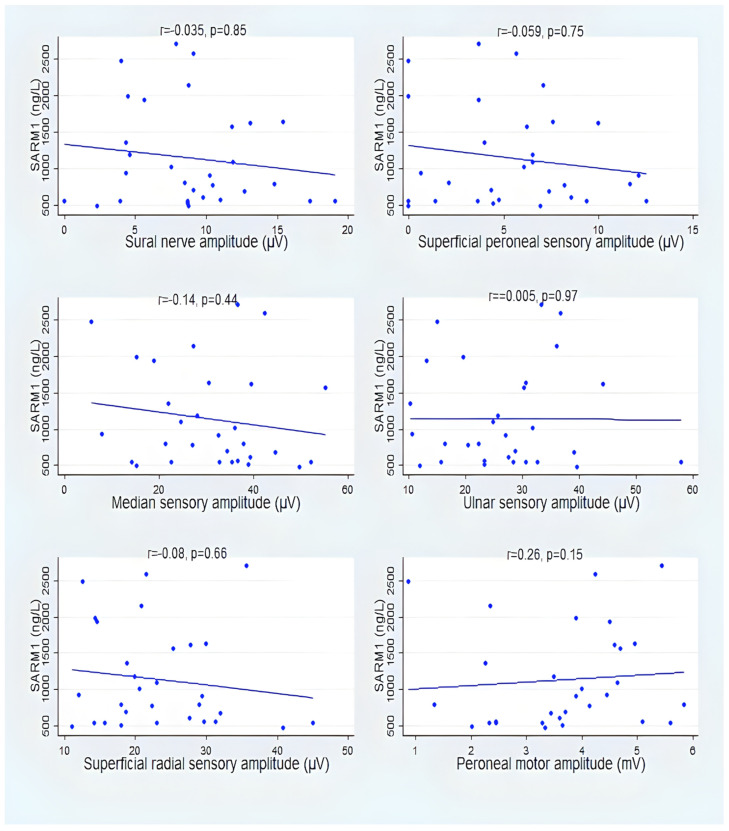
Correlation between SARM1 and amplitude of sensory and motor nerves action potentials in group B.

Figure 4 and Figure 5 show that despite that, there was only one statistically significant correlation between electrodiagnostic studies, median sensory amplitude, and SARM1 in group A. However, there was a consistent direction of negative correlation between SARM1 and the sensory and motor nerve amplitude (see Supplementary Tables S1 and S4 for details).

## 4. Discussion

The present study supports the presence of a correlation between SARM1 serum levels and diabetic neuropathy in T2DM patients when doses of COVID-19 vaccines were considered. SARM1 is considered a negative regulator of the Toll-like receptor 3 and 4 pathways in innate immunity. It has been characterized as a molecule that regulates axonal degeneration. Moreover, it is expressed and functions during neuronal morphogenesis and neurogenesis during the development of the mammalian nervous system [28]. Sundaramoorthy, et al. reported that SARM1 mediates axonal degeneration during neurotropic viral infection [29].

However, the effects of antiviral COVID-19 vaccination on the expression of SARM1 were not investigated. We observed pronounced effects of COVID-19 vaccination on the SARM1 level. Two doses of COVID-19 vaccination increased the SARM1 levels by 119.30%, 76.13%, and 143.13% in the HP group, T2DM group, and T2DMN group, respectively, compared with the median of the single-dose group. Due to the huge surge in the SARM1 level, the correlation was statistically insignificant without consideration of the number of COVID-19 vaccinations. After adjusting for the number of COVID-19 vaccinations, the median serum SARM1 concentration in the T2DMN group was significantly higher than the HP group with a single dose of COVID-19 vaccine (group A). There was no difference in group B, which could be related to the small sample size in group B as well as two vaccination doses causing a surge in the SARM1 levels in all three groups. COVID-19 vaccination doses could negatively or positively interfere with the immune response to COVID-19. SARM1, an adaptor protein released following vaccination, is one of the most important mechanisms responsible for innate antiviral immunity, which promotes axon degeneration [16,30]. Its functions can lead to different immune outcomes, including pro-inflammatory and anti-inflammatory effects [31]. Physiologically and based on our findings, a healthy individual will display increased serum SARM1 levels following COVID-19 vaccination because of the innate immune response. Moreover, SARM1 may be activated in the immune cells by cytotoxic T cells and may promote T cell death, thus either promoting viral infection or preventing excessive inflammation post infection. This mechanism may limit viral spread in virus-infected cells [31]. Furthermore, SARM1 may catalyze NAD+ degradation, which is generally indicative of cell death. The mentioned pathophysiology occurs in neurons and causes axonal [32,33]. SARM1 levels can vary depending on the underlying medical condition [16]. Neurodegenerative diseases, traumatic brain injuries, toxic neuropathy, and peripheral neuropathy increase SARM1 activity in knockout mice [16,34]. Animal studies have indicated that SARM1 gene deficiency decreases DPN in mice and helps to slow axon degeneration, which may be effective in combating the condition [15]. Interestingly, an animal study indicated that vincristine-mediated subacute and chronic axon loss is mediated by SARM1 activation. SARM1 blocking can prevent vincristine-induced peripheral polyneuropathy [32]. Furthermore, SARM1 genetic deletion prevents the development of distal sensory axonal degeneration in metabolic syndrome [35].

Our study demonstrated a consistent correlation between SARM1 and several clinical neuropathy scales, and despite that, it was not statistically significant with all the clinical scales, likely due to the small sample size; however, the correlations were consistently positive. There was a significant positive correlation between the SARM1 level with the MNSI-E and NSS in the participants with a single dose of COVID-19 vaccine after adjusting the numbers of COVID-19 vaccine doses. The participants who had received two doses of the COVID-19 vaccine showed a consistent positive (without reaching the statistical significance) between the SARM1 level and clinical neuropathy scales. While SARM1 did not appear to be associated with the total scores of MNSI and mTCNS, there was a trend towards a higher SARM1 among those with higher total scores of MNSI and mTCNS. MNSI has been proven to correlate with the severity of neuropathy as well as other measures of neuropathy, such as epidermal nerve fiber density [36]. The mTCNS has been shown to be an excellent discriminator between neuropathy and no neuropathy cases [37]. High discrimination capacity has been reported with UENS, with an area under the curve of 0.88 to 0.94 for neuropathy cases [37]. A larger sample size may support this association between SARM1 and clinical neuropathy scales.

SARM1 showed a consistent negative correlation with six different sensory and motor nerve potential amplitudes; however, it reached statistical significance in only the median motor amplitude in group A. The presence of consistent findings may suggest a clinically significant finding despite the fact that statistical significance was not reached, likely due to the small sample size. In the early stages of DPN, diagnosis is challenging because there are no symptoms to be identified. The clinical manifestations of DPN typically occur insidiously. The use of NCS techniques, which can identify subclinical neuropathological changes, makes it possible to diagnose DPN early [38]. In this study, we hypothesized that SARM1, an immune factor, was associated with diabetic neuropathy pathogenesis. Through the destruction of NAD^+^, SARM1 has been shown to promote pathological axonal degeneration; furthermore, SARM1 has an intrinsic ability to cleave NAD+ [39]. As a primary feature of patients with axonal degeneration neuropathies, SNAPs have a markedly reduced amplitude [40]. The amplitude of SNAPs has a strong correlation with peripheral neuropathy. SNAPs abnormalities are most prevalent in the median nerve, suggesting that diabetes affects this nerve first [38]. Also, this finding, considering the impact of a limited sample size, is in agreement with a previous study that found that loss of sensory nerve action potential amplitude occurs as a result of SARM1-dependent processes in the tail nerve in a mouse paclitaxel model of chemotherapy-induced peripheral neuropathy [41].

By considering the potential impact of the COVID-19 vaccine on SARM1 levels, researchers can better understand any observed differences or lack thereof between study groups. It is crucial to recognize that SARM1 levels may be transient and can vary based on the timing of measurements. Therefore, it is necessary to account for these factors when interpreting study results.

Moreover, the potential neurological complications associated with the COVID-19 vaccine should not be overlooked. Cases of transverse myelitis, Guillain–Barré syndrome, encephalopathy, and Bell’s palsy have been reported following vaccination [20,21]. These complications have implications for the interpretation of study results and should be taken into consideration in discussions surrounding the vaccine’s safety and efficacy.

Emphasizing the significance of considering the impact of the COVID-19 vaccine on SARM1 levels and its transient nature, as well as the timing of measurements, is crucial for accurately interpreting study results. Additionally, it is important to acknowledge and address the potential neurological complications associated with the vaccine. Furthermore, due to the challenges of obtaining a large sample size during a COVID-19 pandemic or future pandemics, it is vital to share this result study with other scientists.

### Strengths and Limitations

To improve the quality of the data, we removed specific confounding factors by dividing the patients into major groups according to the COVID-19 vaccine, which may affect the power of the study to detect differences between the groups. We interviewed all participants to avoid misunderstandings and increase the validity of the responses.

The main limitation of the present study is its small sample size. However, we thought this sample size showed a significant influence of the COVID-19 vaccination on the SARM1 level, which carries a lot of significance and should guide selection criteria and analysis in future studies. Additionally, because of logistical reasons, including the COVID-19 pandemic, we were unable to increase the number of patients in our study. Other limitations include the single-institution nature of the study and the absence of skin biopsy for epidermal nerve fiber density (ENFD) measurements. On the other hand, our cohort of patients was clinically characterized through many clinical scales and electrodiagnostic studies that have been demonstrated to be strongly correlated with ENFD.

## 5. Conclusions

In this study, SARM1 may have potential as a biomarker for the early detection of diabetic peripheral neuropathy in type 2 diabetes patients. However, it is important to acknowledge certain limitations. The changes in SARM1 levels observed following COVID-19 vaccination are interesting, but the clinical significance and long-term implications of these changes are still unclear. To validate our findings and further explore the role of SARM1 in peripheral neuropathies, future research should involve larger sample sizes and include pre-vaccination measurements. In our study, COVID-19 vaccination was found to cause changes in serum SARM1 levels in both diabetic and non-diabetic individuals. We consistently observed a positive correlation (although only a few reached statistical significance) between SARM1 levels and clinical neuropathy scales, as well as a consistent negative correlation (with only one reaching statistical significance) between SARM1 and sensory nerve amplitudes. To gain a better understanding of the precise role of SARM1 in axonal function and structural degeneration, future studies should consider larger sample sizes and investigate epidermal fiber density using skin biopsy. Considering its potential, SARM1 could be a promising target for the development of therapeutic interventions aimed at improving the treatment of peripheral neuropathies.

## Figures and Tables

**Table 1:** Demographic and clinical characteristics among the major study groups.

Groups	HP (*n* = 30) (37.5%)	T2DM (*n* = 20) (25%)	T2DMN (*n* = 30) (37.5%)
Variables	**Median (IQR) (25–75)**		
Age (years)	46 (42.75–54)	54.5 (46–57.75)	51.5 (47.7–56.25)
Sex, female, n (%)	17 (56.7)	9 (45)	15 (50)
DV (month)	3 (2–4)	2.5 (2–3)	3 (2–4)
DD (year)	-	5.50 (3.25–14)	12.5 (10–18.25)
HbA1c (%)	5.4 (5.1–5.5)	7 (6.1–7.72)	7.45 (6.6–8.05)
FBG (mmol/L)	5.15 (4.9–5.4)	6.65 (5.9–8.65)	8.05 (6.3–10.05)
SARM1 (ng/L)	437 (375.03–717.8)	521.4 (441.2–784.78)	495.3 (404.2–590.08)
MNSI-ar (n) (sum score)	0.5 (0–3.5)	1.5 (0–11)	5.50 (2–9.5)
mTCNS (n) (sum score)	0.5 (0–5.5)	1 (0–14.75)	6 (1–11.25)
UENS (n) (sum score)	0.00 (0.00–0.00)	0.00 (0.00–8.75)	3 (0–10)
NSS (n)	0.00 (0.00–5.50)	0.00 (0–6)	2 (0–8)
NDS (n) (sum score)	0.00 (0.00–0.00)	0.00 (0.00–6.25)	2 (0–7.25)
MS AMP (mV)	39.45 (35.77–52.54)	33.17 (25.22–40.72)	17.8 (9.79–27.65)
US AMP (mV)	26.95 (22.19–38)	23.22 (15.98–28.63)	14.1 (7.06–26.05)
SS AMP (mV)	13.37 (9.63–15.65)	9.32 (8.13–11.76)	5.32 (1.72–8.81)
SPS AMP (mV)	7.82 (6.43–10.12)	6.02 (3.88–10.30)	1.54 (0.00–5.30)

Note: DV refers to the duration of the vaccine, which is calculated from the date of the last dose of the COVID-19 vaccine. Abbreviations: IQR: interquartile range; CI: confidence interval; G1: healthy participants; G2: type 2 diabetes mellitus; G3: T2DM with neuropathy; MW-U: Mann–Whitney U test; KWT: Kruskal–Wallis test; DD: duration of diseases; FBG: fasting blood glucose; HbA1c: hemoglobin A1c; SARM1: sterile-a and Toll/interleukin-1 receptor domain-containing protein 1; Q: questionnaire; E: examination; NSS: Neuropathy Symptoms Score; MNSI: Michigan Neuropathy Screening Instrument; mTCNS: Modified Toronto Clinical Neuropathy Score; UENS: Utah Early Neuropathy Scale examination; NDS: neuropathy disability score; AMP: amplitude; MS: median sensory; US: ulnar sensory; SS: sural sensory; SPS: superficial peroneal sensory; IQR: interquartile range; *p*-value < 0.05.

**Table 2:** Comparison of demographic and clinical characteristics among the major study groups.

Groups	G1 and 2			G1 and 3			G2 and 3			All Groups
	MW-U	95% CI		MW-U	95% CI		MW-U	95% CI		KWT
Variables	***p*-value**	**Lower Bound**	**Upper Bound**	***p*-value**	**Lower Bound**	**Upper Bound**	***p*-value**	**Lower Bound**	**Upper Bound**	***p*-value**
Age (years)	0.025	0.022	0.028	0.032	0.028	0.035	0.785	0.777	0.793	0.037
DV (month)	0.295	0.286	0.303	0.323	0.314	0.332	0.986	0.984	0.988	0.484
DD (year)	<0.001	<0.001	<0.001	<0.001	<0.001	<0.001	0.014	0.012	0.016	<0.001
HbA1c (%)	<0.001	<0.001	<0.001	<0.001	<0.001	<0.001	0.254	0.245	0.262	<0.001
FBG (mmol/L)	<0.001	<0.001	<0.001	<0.001	<0.001	<0.001	0.147	0.140	0.154	<0.001
SARM1 (ng/L)	0.193	0.185	0.201	0.567	0.557	0.577	0.334	0.325	0.343	0.381
MNSI-ar (n) (sum score)	<0.001	<0.001	0.001	<0.001	<0.001	<0.001	<0.001	<0.001	<0.001	<0.001
mTCNS (n) (sum score)	0.002	0.001	0.003	<0.001	<0.001	<0.001	<0.001	<0.001	<0.001	<0.001
UENS (n) (sum score)	0.007	0.005	0.008	<0.001	<0.001	<0.001	<0.001	<0.001	<0.001	<0.001
NSS (n)	0.021	0.018	0.024	<0.001	<0.001	<0.001	<0.001	<0.001	<0.001	<0.001
NDS (n) (sum score)	0.024	0.021	0.027	<0.001	<0.001	<0.001	<0.001	<0.001	<0.001	<0.001
MS AMP (mV)	0.005	0.004	0.007	<0.001	<0.001	<0.001	<0.001	<0.001	<0.001	<0.001
US AMP (mV)	0.015	0.013	0.017	<0.001	<0.001	<0.001	0.035	0.031	0.038	<0.001
SS AMP (mV)	0.018	0.015	0.020	<0.001	<0.001	<0.001	0.002	0.001	0.003	<0.001
SPS AMP (mV)	0.103	0.097	0.109	<0.001	<0.001	<0.001	<0.001	<0.001	<0.001	<0.001

Note: DV refers to the duration of the vaccine, which is calculated from the date of the last dose of the COVID-19 vaccine. Abbreviations: IQR: interquartile range; CI: confidence interval; G1: healthy participants; G2: type 2 diabetes mellitus; G3: T2DM with neuropathy; MW-U: Mann–Whitney U test; KWT: Kruskal–Wallis test; DD: duration of diseases; FBG: fasting blood glucose; HbA1c: hemoglobin A1c; SARM1: sterile-a and Toll/interleukin-1 receptor domain-containing protein 1; Q: questionnaire; E: examination; NSS: Neuropathy Symptoms Score; MNSI: Michigan Neuropathy Screening Instrument; mTCNS: Modified Toronto Clinical Neuropathy Score; UENS: Utah Early Neuropathy Scale examination; NDS: neuropathy disability score; AMP: amplitude; MS: median sensory; US: ulnar sensory; SS: sural sensory; SPS: superficial peroneal sensory; IQR: interquartile range; *p*-value < 0.05.

**Table 3:** Demographic and clinical characteristics among the subgroups stratified by the number of previous COVID-19 vaccinations: group A (a single dose of COVID-19 vaccine) and group B (two doses of COVID-19 vaccine).

Groups	Group A				Group B			
	HP (*n* = 18)	T2DM (*n* = 10)	T2DMN (*n* = 22)	*p*-Value	HP (*n* = 12)	T2DM (*n* = 10)	T2DMN (*n* = 8)	*p*-Value
Variables	** Median ** **(** ** IQR) (25–75) **		**KWT**		** Median ** **(** ** IQR) (25–75) **		**KWT**	
Age (years)	46.5 (42–53.25)	53 (44.75–58)	52 (46.75–57.25)	0.173	45.5 (44–54.75)	55 (46.5–57.5)	50.5 (48–53.75)	0.159
Sex, female, *n* (%)	12 (66.7)	6 (54.5)	11 (50)	-	5 (41.7)	3 (33.3)	4 (50)	-
DV (month)	3 (2–4)	3.5 (2–4.25)	3 (1.75–4)	0.589	3.5 (2.25–4.75)	3 (2–3)	3.5 (2–4.75)	0.240
DD (year)	0.00 (0–0) ^α, €^	4 (1–8) ^¥^	13 (8.5–18.25)	0.000	0.00 (0–0) ^α, €^	8 (3.75–15)	12.5 (10–22.25)	0.000
HbA1c (%)	5.4 (5.1–5.5) ^α, €^	7 (5.97–7.5)	7.15 (6.57–8)	0.000	5.3 (5.22–5.5) ^α, €^	7.8 (6.4–8.55)	7.65 (6.87–9.22)	0.000
FBG (mmol/L)	5.15 (4.9–5.52) ^α, €^	6.5 (5.4–7.8)	7.85 (6.37–10)	0.000	5.15 (4.82–5.4) ^α, €^	7.45 (6.2–9.97)	8.95 (6.65–10.2)	0.000
SARM1 (ng/L)	384.8 (362.55–429.25) ^α, €^	443.7 (409.2–502.8)	468.1 (388.92–520.07)	0.022	843.6 (619.2–1597.02)	778.6 (538.6–1368.9)	1138.1 (497.5–2349.77)	0.803
MNSI-ar (*n*) (sum score)	0.00 (0–1.25) ^α, €^	2 (1.25–4) ^¥^	10.25 (7.37–12.62)	0.000	0.00 (0–0.75) ^€^	2 (0–2.62) ^¥^	9.25 (7.25–11)	0.000
mTCNS (*n*) (sum score)	0.00 (0–0.5) ^α, €^	1.75 (0–5) ^¥^	11.5 (8–22)	0.000	0.00 (0–0) ^€^	0.5 (0–5.1) ^¥^	11 (6.12–18.25)	0.000
UENS (*n*)	0.00 (0–0) ^€^	0.00 (0–0.5) ^¥^	6 (2–14.75)	0.000	0.00 (0–0) ^€^	0.00 (0–2.25) ^¥^	10 (8–10.75)	0.000
NSS (*n*)	0.00 (0–0) ^α, €^	0.00 (0–5.5) ¥	8(6.75–9)	0.000	0.00 (0–0) ^€^	0.00 (0–0)	6 (5.25–6.75)	0.000
NDS (*n*)	0.00 (0–0) €	0.00 (0–0) ¥	4 (2–7.5)	0.000	0.00 (0–0) ^α, €^	0.00 (0–2) ¥	8 (7.25–9)	0.000
MS AMP (mV)	42.52 (37.07–55.97) ^€^	39.6 (31.52–49.5) ¥	17.8 (9.78–27.65)	0.000	38.65 (33.3–44.06) ^α, €^	27.3 (20.82–35.13)	18.72 (9.82–33.17)	0.003
US AMP (mV)	34.07 (23.76–46.80) ^€^	28.6 (19.9–31.3) ¥	14.87 (6.82–27.67)	0.000	22.87 (20.35–28.15) ^α^	17.07 (14.2–25.36)	13.65 (7.51–24.15)	0.052
SS AMP (mV)	14.72 (11.30–16.70) ^€^	10 (8.3–14.5) ¥	6.37 (0.00–10.4)	0.000	11.05 (9.11–14.38) ^€^	8.7 (5.4–11.22) ¥	4.37 (2.77–7.05)	0.001
SPS AMP (mV)	8.07 (7.02–10.12) ^€^	8.05 (4.3–14.1) ¥	2.85 (0.00–6.2)	0.000	7.5 (5.8–11.2) ^€^	5.65 (3.31–7.38) ¥	0.32 (0.00–3.92)	0.002

α: *p* < 0.05 for G1 versus G2; €: *p* < 0.05 for G1 versus G3; ¥: *p* < 0.05 for G2 versus G3. Note: DV refers to the duration of the vaccine, which is calculated from the date of the last dose of the COVID-19 vaccine. Abbreviations: IQR: interquartile range; CI: confidence interval; G1: healthy participants; G2: type 2 diabetes mellitus; G3: T2DM with neuropathy; MW-U: Mann–Whitney U test; KWT: Kruskal–Wallis test; DD: duration of diseases; FBG: fasting blood glucose; HbA1c: hemoglobin A1c; SARM1: sterile-a and Toll/interleukin-1 receptor domain-containing protein 1; Q: questionnaire; E: examination; NSS: Neuropathy Symptoms Score; MNSI: Michigan Neuropathy Screening Instrument; mTCNS: Modified Toronto Clinical Neuropathy Score; UENS: Utah Early Neuropathy Scale examination; NDS: neuropathy disability score; AMP: amplitude; MS: median sensory; US: ulnar sensory; SS: sural sensory; SPS: superficial peroneal sensory; IQR: interquartile range; *p*-value < 0.05.

**Table 4:** Correlation between SARM1 and demographic and clinical characteristics among all participants.

Variables	Spearman’s rho	Significance (2-tailed)	95% Confidence Interval	
			Lower	Upper
**Age (years)**	0.083	0.464	−0.146	0.303
**DV (month)**	−0.028	0.802	−0.253	0.199
**DD (year)**	0.108	0.340	−0.121	0.326
**HbA1c (%)**	0.094	0.409	−0.135	0.313
**FBG (mmol/L)**	0.103	0.362	−0.126	0.322
**MNSI-ar (n) (sum score)**	−0.022	0.848	−0.247	0.205
**MNSIq (n)**	−0.054	0.636	−0.276	0.174
**MNSIe (n)**	0.036	0.753	−0.192	0.260
**mTCNS (n) (sum score)**	−0.018	0.873	−0.243	0.209
**mTCNS q (n)**	−0.094	0.409	−0.313	0.135
**mTCNSe (n)**	0.120	0.289	−0.109	0.337
**UENS** **(n) (sum score)**	0.108	0.342	−0.121	0.326
**NSS (n)**	−0.013	0.910	−0.238	0.214
**NDS** **(n) (sum score)**	0.135	0.231	−0.093	0.351
**MS AMP (mV)**	−0.214	0.057	−0.419	0.013
**US AMP (mV)**	−0.242	0.031	−0.443	−0.016
**RS AMP (mV)**	−0.228	0.042	−0.431	−0.002
**SS AMP (mV)**	−0.152	0.179	−0.365	0.077
**SPS AMP (mV)**	−0.114	0.316	−0.331	0.115
**PM AMP (mV)**	0.212	0.059	−0.014	0.418

Note: DV refers to the duration of the vaccine, which is calculated from the date of the last dose of the COVID-19 vaccine. Abbreviations: CI: confidence interval; DD: duration of diseases; FBG: fasting blood glucose; HbA1c: hemoglobin A1c; SARM1: sterile-a and Toll/interleukin-1 receptor domain-containing protein 1; Q: questionnaire; E: examination; NSS: Neuropathy Symptoms Score; MNSI: Michigan Neuropathy Screening Instrument; mTCNS: Modified Toronto Clinical Neuropathy Score; UENS: Utah Early Neuropathy Scale examination; NDS: neuropathy disability score; AMP: amplitude; MS: median sensory; US: ulnar sensory; SS: sural sensory; SPS: superficial peroneal sensory; *p*-value < 0.05.

**Table 5:** Correlation between SARM1 and demographic and clinical characteristics among the groups stratified by the number of previous COVID-19 vaccinations: group A (a single dose of COVID-19 vaccine) and group B (two doses of COVID-19 vaccine).

Groups	Group A				Group B			
Variables	**Spearman’s rho**	**Significance (2-tailed)**	**95%** **CI**		**Spearman’s rho**	**Significance (2-tailed)**	**95%** **CI**	
			**Lower**	**Upper**			**Lower**	**Upper**
Age (years)	0.156	0.280	−0.136	0.423	0.082	0.667	−0.297	0.438
DV (month)	−0.215	0.133	−0.472	0.076	0.307	0.099	−0.071	0.608
DD (year)	0.310	0.029	0.026	0.547	−0.101	0.596	−0.454	0.280
HbA1c (%)	0.267	0.061	−0.021	0.514	−0.100	0.598	−0.453	0.280
FBG (mmol/L)	0.201	0.163	−0.091	0.460	0.012	0.949	−0.359	0.380
MNSI-ar (n) (sum score)	0.240	0.093	−0.049	0.493	0.132	0.486	−0.250	0.479
MNSIq (n)	0.198	0.168	−0.093	0.458	0.190	0.316	−0.194	0.523
MNSIe (n)	0.288	0.043	0.002	0.530	0.001	0.997	−0.369	0.371
mTCNS (n) (sum score)	0.217	0.130	−0.074	0.474	0.146	0.440	−0.236	0.490
mTCNS q (n)	0.175	0.223	−0.117	0.439	0.038	0.841	−0.337	0.402
mTCNSe (n)	0.267	0.060	−0.020	0.514	0.340	0.066	−0.034	0.631
UENS (n) (sum score)	0.231	0.107	−0.059	0.485	0.333	0.072	−0.042	0.626
NSS (n)	0.327	0.021	0.045	0.560	0.176	0.353	−0.208	0.512
NDS (n) (sum score)	0.276	0.053	−0.011	0.521	0.293	0.116	−0.086	0.598
MS AMP (mV)	−0.328	0.020	−0.562	−0.047	−0.145	0.445	−0.489	0.238
US AMP (mV)	−0.085	0.557	−0.362	0.206	−0.162	0.391	−0.502	0.221
RS AMP (mV)	−0.062	0.667	−0.342	0.228	−0.081	0.670	−0.438	0.298
SS AMP (mV)	−0.099	0.495	−0.374	0.193	−0.035	0.853	−0.400	0.339
SPS AMP (mV)	−0.069	0.635	−0.348	0.222	−0.059	0.755	−0.420	0.317
PM AMP (mV)	−0.048	0.738	−0.330	0.241	0.264	0.158	−0.117	0.578

Note: DV refers to the duration of the vaccine, which is calculated from the date of the last dose of the COVID-19 vaccine. Abbreviations: CI: confidence interval; DD: duration of diseases; FBG: fasting blood glucose; HbA1c: hemoglobin A1c; SARM1: sterile-a and Toll/interleukin-1 receptor domain-containing protein 1; Q: questionnaire; E: examination; NSS: Neuropathy Symptoms Score; MNSI: Michigan Neuropathy Screening Instrument; mTCNS: Modified Toronto Clinical Neuropathy Score; UENS: Utah Early Neuropathy Scale examination; NDS: neuropathy disability score; AMP: amplitude; MS: median sensory; US: ulnar sensory; SS: sural sensory; SPS: superficial peroneal sensory; *p*-value < 0.05.

## Data Availability

The data presented in this study are available upon request from the corresponding author.

## Supplementary Materials

The following supporting information can be downloaded at: https://www.mdpi.com/article/10.3390/vaccines12020209/s1. Table S1: Correlation between SARM1, clinical characteristics and neuropathy scales among the whole cohort and the groups stratified by the number of previous COVID-19 vaccinations. Only correlations achieving *p* < 0.05 were considered; Table S2: SARM1 level correlation with healthy and diabetic participants with and without neuropathy groups; Table S3: SARM1 level correlation with healthy, duration (years) ≤ 3, and Duration (years) > 3; Table S4: Correlation between SARM1, clinical characteristics, neuropathy scales and NCSs among the whole cohort and the groups stratified by the number of previous COVID-19 vaccinations.
