# Supplementary material for: Serum SARM1 Levels and Diabetic Peripheral Neuropathy in Type 2 Diabetes: Correlation with Clinical Neuropathy Scales and Nerve Conduction Studies and Impact of COVID-19 vaccination
Source: Vaccines (Basel). 2024 Feb 17;12(2):209. doi: 10.3390/vaccines12020209 (PMC10892204; doi:10.3390/vaccines12020209)
Supplement: Supplementary file 1 [file vaccines-12-00209-s001.zip › vaccines-2825292-supplementary.pdf]

Table S1. Correlation between SARM1, clinical characteristics and neuropathy scales among the whole cohort and the groups stratified by the number of previous COVID-19 vaccinations. Only correlations achieving p<0.05 were considered. Supplementary Tables

Predictors	Groups	Correlation coefficient	95% CI	P-value
Duration of diabetes	Whole cohort (n=80)	0.099	-0.072-0.302	0.386
	Group A (n=50)	0.314	0.037-0.534	0.028
	Group B (n=30)	-0.101	-0.521-0.327	0.598
MNSI- E (physical examination only)	Whole cohort (n=80)	0.036	-0.194-0.327	0.753
	Group A (n=50)	0.288	-0.015-0.560	0.043
	Group B (n=30)	-0.001	0-0.394-0.419	0.997
NSS	Whole cohort (n=80)	-0.013	-0.240-0.215	0.910
	Group A (n=50)	0.327	-0.093-0.556	0.021
	Group B (n=30)	0.176	-0.207-0.626	0.353
MS AMP	Whole cohort (n=80)	-0.205	-0.392-0.027	0.070
	Group A (n=50)	-0.336	-0.538-(-0.027)	0.018
	Group B (n=30)	-0.145	-0.572-0.283	0.445
US AMP	Whole cohort (n=80)	-0.242	-0.461-0.024	0.031
	Group A (n=50)	-0.085	-0.370-0.200	0.557
	Group B (n=30)	-0.162	-0.531-0.180	0.391

Abbreviation: SARM1: sterile-a and Toll/interleukin-1 receptor domain-containing protein 1; DD: duration of diseases; MNSI-E: Michigan Neuropathy Screening Instrument examination; NSS: Neuropathy Symptoms Score; AMP: amplitude; MS: median sensory; US: ulnar sensory; P-value < 0.05.

Table S2. SARM1 level correlation with healthy and diabetic participants with and without neuropathy groups. Supplementary Tables

Model	Groups	OR	95% CI	P-value
Crude (Unadjusted)	HP (n=30)	Reference		
	T2DM (n=20)	1.000	0.999-1.001	0.945
	T2DMN (n=30)	1.001	1.000-1002	0.214
COVID-19 vaccine (Adjusted)				
A single dose of the vaccine	HP (n=18)	Reference		
	T2DM (n=10)	1.010	0999-1.021	0.088
	T2DMN (n=22)	1.013	1.003-1.023	0.013
Two doses of the vaccine	HP (n=12)	Reference		
	T2DM (n=10)	1.000	0.999-1.001	0.737
	T2DMN (n=8)	1.001	0.999-1.002	0.406

Dependent variable: T2DMN (three levels: "HP" "T2DM," and "T2DMN"). The reference category of the dependent variable was "Healthy participants". Independent variable: SARM1. The confounder factors were adjusted by the numbers of COVID-19 vaccine doses

COVID-19: coronavirus disease 2019; T2DM: type 2 diabetes mellitus; T2DMN: T2DM with neuropathy; OR: odds ratio; 95% CI: 95% confidence interval; and HP: healthy participants. P-value < 0 05.

Table S3. SARM1 level correlation with healthy, duration (years) ≤ 3, and Duration (years) > 3. Supplementary Tables

Model	Groups	OR	95% CI	P-value
Crude (Unadjusted)	HP	Reference		

	Duration (years) ≤ 3	1.000	0.998-1.002	0.807
	Duration (years) > 3	1.000	0.999-1002	0.489
COVID-19 vaccine (Adjusted)				
A single dose of the vaccine	HP	Reference		
	Duration (years) ≤ 3	1.016	1.003-1.029	0.015
	Duration (years) > 3	1.011	1.001-1.021	0.027
Two doses of the vaccine	HP	Reference		
	Duration (years) ≤ 3	1.000	0.997-1.002	0.809
	Duration (years) > 3	1.001	0.999-1.001	0.721

Dependent variable: Duration of T2DM (years) > 3 (three levels: "Healthy participants" "duration of T2DM (years) ≤ 3," and "Duration (years) > 3"). The reference category of the dependent variable was "HP", Independent variable: SARM1. The confounder factors were adjusted by the numbers of COVID-19 vaccine doses

COVID-19: coronavirus disease 2019; OR: odds ratio; 95% CI: 95% confidence interval; and HP: healthy participants. P-value < 0 05.

Table S4. Correlation between SARM1, clinical characteristics, neuropathy scales and NCSs among the whole cohort and the groups stratified by the number of previous COVID-19 vaccinations.

Supplementary Tables

Variables	Groups	Spearman's rho	P value	95% CI	
				Lower	Lower
**Age (years)**	Whole cohort (n=80)	0.083	0.464	-0.146	0.303

	Group A (n=50)	0.156	0.280	-0.136	0.423
	Group B (n=30)	0.082	0.667	-0.297	0.438
**DV (month)**	Whole cohort (n=80)	-0.028	0.802	-0.253	0.199
	Group A (n=50)	-0.215	0.133	-0.472	0.076
	Group B (n=30)	0.307	0.099	-0.071	0.608
**DD (year)**	Whole cohort (n=80)	0.099	0.386	-0.132	0.319
	Group A (n=50)	0.314	0.028	0.028	0.553
	Group B (n=30)	-0.101	0.596	-0.454	0.280
**HbA1c (%)**	Whole cohort (n=80)	0.094	0.409	-0.135	0.313
	Group A (n=50)	0.267	0.061	-0.021	0.514
	Group B (n=30)	-0.100	0.598	-0.453	0.280
**FBG (mmol/L)**	Whole cohort (n=80)	0.103	0.362	-0.126	0.322
	Group A (n=50)	0.201	0.163	-0.091	0.460
	Group B (n=30)	0.012	0.949	-0.359	0.380
**MNSI (n) (sum score)**	Whole cohort (n=80)	-0.022	0.848	-0.247	0.205
	Group A (n=50)	0.240	0.093	-0.049	0.493
	Group B (n=30)	0.132	0.486	-0.250	0.479
**MNSI-Q (n)**	Whole cohort (n=80)	-0.054	0.636	-0.276	0.174
	Group A (n=50)	0.198	0.168	-0.093	0.458
	Group B (n=30)	0.190	0.316	-0.194	0.523
**MNSI-E (n)**	Whole cohort (n=80)	0.036	0.753	-0.192	0.260
	Group A (n=50)	0.288	0.043	0.002	0.530
	Group B (n=30)	0.001	0.997	-0.369	0.371
**mTCNS (n) (sum score)**	Whole cohort (n=80)	-0.018	0.873	-0.243	0.209
	Group A (n=50)	0.217	0.130	-0.074	0.474
	Group B (n=30)	0.146	0.440	-0.236	0.490

**mTCNS-Q (n)**	Whole cohort (n=80)	-0.094	0.409	-0.313	0.135
	Group A (n=50)	0.175	0.223	-0.117	0.439
	Group B (n=30)	0.038	0.841	-0.337	0.402
**mTCNS-E (n)**	Whole cohort (n=80)	0.120	0.289	-0.109	0.337
	Group A (n=50)	0.267	0.060	-0.020	0.514
	Group B (n=30)	0.340	0.066	-0.034	0.631
**UENS (n)**	Whole cohort (n=80)	0.108	0.342	-0.121	0.326
	Group A (n=50)	0.231	0.107	-0.059	0.485
	Group B (n=30)	0.333	0.072	-0.042	0.626
**NSS (n)**	Whole cohort (n=80)	-0.013	0.910	-0.238	0.214
	Group A (n=50)	0.327	0.021	0.045	0.560
	Group B (n=30)	0.176	0.353	-0.208	0.512
**NDS (n)**	Whole cohort (n=80)	0.135	0.231	-0.093	0.351
	Group A (n=50)	0.276	0.053	-0.011	0.521
	Group B (n=30)	0.293	0.116	-0.086	0.598
**DNS (n)**	Whole cohort (n=80)	-0.077	0.497	-0.298	0.152
	Group A (n=50)	0.212	0.140	-0.079	0.469
	Group B (n=30)	0.037	0.846	-0.338	0.401
**MS AMP (mV)**	Whole cohort (n=80)	-0.214	0.057	-0.419	0.013
	Group A (n=50)	-0.328	0.020	-0.562	-0.047
	Group B (n=30)	-0.145	0.445	-0.489	0.238
**US AMP (mV)**	Whole cohort (n=80)	-0.242	0.031	-0.443	-0.016
	Group A (n=50)	-0.085	0.557	-0.362	0.206
	Group B (n=30)	-0.162	0.391	-0.502	0.221
**SS AMP (mV)**	Whole cohort (n=80)	-0.152	0.179	-0.365	0.077
	Group A (n=50)	-0.099	0.495	-0.374	0.193

	Group B (n=30)	-0.035	0.853	-0.400	0.339
SPS AMP (mV)	Whole cohort (n=80)	-0.114	0.316	-0.331	0.115
	Group A (n=50)	-0.069	0.635	-0.348	0.222
	Group B (n=30)	-0.059	0.755	-0.420	0.317

Abbreviation: CI: Confidence Interval; DD: duration of diseases; FBG: fasting blood glucose; HbA1c: hemoglobin A1c; SARM1: sterile-a and Toll/interleukin-1 receptor domain-containing protein 1; Q: Questionnaire; E: examination; NSS: Neuropathy Symptoms Score; MNSI: Michigan Neuropathy Screening Instrument; mTCNS: Modified Toronto Clinical Neuropathy Score; UENS: Utah Early Neuropathy Scale examination; NDS, neuropathy disability score – examination; AMP: amplitude; MS: median sensory; US: ulnar sensory; SS: sural sensory; SPS: superficial peroneal sensory; mV: millivolt; P-value < 0.05.
